# Low Vision Services Provision throughout NHS Trusts in the UK

**DOI:** 10.22599/bioj.293

**Published:** 2023-08-08

**Authors:** Charlotte Joy Codina, Martin Rhodes

**Affiliations:** 1 University of Sheffield, UK; 2 Sheffield Teaching Hospitals Trust, UK

**Keywords:** Low Vision, Inequality, vision impaired, rehabilitation, NHS workforce

## Abstract

**Background/Aims:**

The aim of this study was to understand the picture of low vision service provision within NHS Trusts in the United Kingdom, for children and adults.

**Method:**

A survey was distributed to all members of the British and Irish Orthoptic Society (BIOS) and to all Eye Clinic Liaison Officers (ECLOs) through the Royal National Institute for the Blind (RNIB). The survey was also directly emailed to Orthoptic contacts of all 238 Trusts/Health Boards, which covered the four nations of the United Kingdom. The survey asked whether their Trust had a clinical low vision service, which professions were involved in leading and working within it, where it was based, and whether provision was offered to children, adults, or both.

**Results:**

In the United Kingdom (UK), 117 out of 238 (49%) Trusts responded. Of these responders, 94% had a level of Trust-delivered low vision service provision; 90% had services for adults; 83% had services for children; and 79% had services for both adults and children. Service accessibility for patients of all ages had regional and national variation.

**Conclusion:**

Significant variation was found in low vision service provision throughout the UK, with some regions having no NHS-delivered provision for either children, adults, or both. This calls for further research to gain a more comprehensive understanding of low vision service provision and remove inequalities in provision, access and resourcing, aiming to ensure equitable access for all.

## Introduction

Over the last 50 years, the directive and aims of low vision care have changed, from assisting people with a vision impairment (VI) to read with the use of magnifiers, to a holistic rehabilitation process involving the multi-disciplinary team and allied services (Ryan et al. 2014).

It is estimated that more than two million people in the UK are living with a VI (RNIB, 2021). This number is ever increasing, with the ageing population and epidemic of diabetes being just some of the reasons for this (Government Office for Science, 2016; Office for National Statistics, 2015). This picture not only includes people who are registered as vision impaired, but also includes individuals waiting for treatment, those whose sight could be improved, those who have not registered, and people whose VI is not at a level that allows them to register (RNIB, 2021).

VI has significant cost implications for the UK, with an estimated total cost of around £28 billion per year Luengo-Fernandez, Leal and Gray (2012). This includes the indirect costs of low vision such as lower employment rates or the cost associated with the provision of informal care. Additionally, it includes the total cost of the reduced wellbeing and health associated with low vision. Low vision is highly associated with depression in the older adults (Virgili et al. 2022) and in younger and middle-aged adults (Brunes et al. 2020). The holistic service approach of the low vision NHS service is imperative when considering these figures: more than 4 in 10 people attending low vision clinics are suffering from symptoms of clinical depression and 31% of blind and partially sighted people were found to be rarely or never optimistic about the future (RNIB, 2021).

Large disparities in low vision service provision for children and adults occur within the UK, with different healthcare professions involved, variable settings, and regional variability in patient access to services and the level of resources they provide. The Low Vision Service Model Evaluation (LOVSME) project (Dickinson et al. 2011) highlighted this significant disparity within low vision service provision across the UK and the lack of standardisation of a delivery model, and it appears that this disparity has existed for some time. In 2002, Culham et al. reported that the distribution of low service provision was geographically uneven and appeared scarce in some regions. Culham also compared the provision with the probable number of people with a VI in the UK and highlighted inadequacies in distribution, magnitude, and coordination of low vision service delivery. This is not just a problem in the UK. The provision and funding of low vision rehabilitation (LVR) has also been reported as very variable in other parts of the world such as Canada in 2016, when the solution of a new integrated care model was proposed (Leat et al. 2016), and Trinidad and Tobago, with Joshi, Persad and Farnon (2021) calling for a unified and comprehensive approach to low vision services.

The low vision service can play a vital role in the habilitation and rehabilitation of people with VI, yet access to this service is far from equitable throughout the UK. This project was developed to document wide low vision service provision throughout the UK as reported by Orthoptists and Eye Clinic Liaison Officers (ECLOs) nationwide, as well as to discuss the implications of the findings.

## Methods

A Google form survey was distributed to all members of the British and Irish Orthoptic Society (BIOS) and to all ECLOs through the Royal National Institute for the Blind (RNIB). An introductory paragraph explained that by completing the questionnaire, participants consented to their data being used anonymously and for the purpose of research. This research adhered to the declaration of Helsinki. To improve the response rate, we directly emailed Orthoptic contacts for 238 Trusts/Health Boards in total, providing them with a direct link to the survey. These were 209 Trusts in England (219 boards exist in England, but ten were excluded as ambulance Trusts), three NHS Trusts and seven Health Boards in Wales (totalling 10), five Trusts in Northern Ireland, and 14 Health Boards in Scotland.

**Figure 1:**
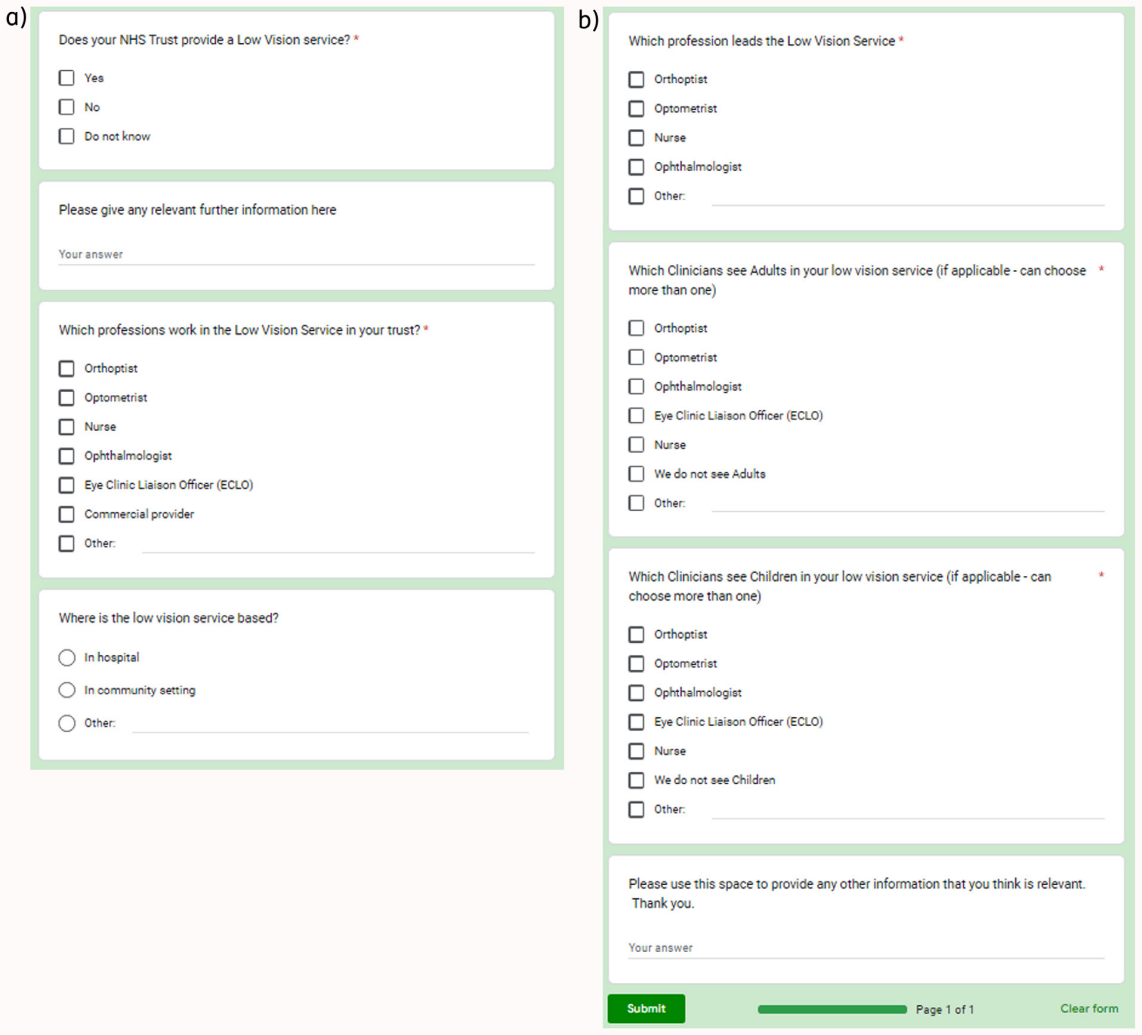
Shows the questionnaire distributed to Orthoptists and ECLOs.

Anonymous data was gathered from the Google form results, exported to Excel and analysed. Graphical and descriptive statistics were applied. Please see Figure 1(a&b) for the questionnaire distributed to Orthoptists and ECLOs.

## Results

### How many Trusts responded?

There were 117 Trusts who responded, signifying a 49% response rate (we received 176 responses – a total of 59 were removed as either duplicates or from outside the UK). For England, 104 Trusts responded (50% response rate, two of which were paediatric specialist units). For Wales, responses were received from six out of ten Trusts (60% response rate). For Scotland, responses were received from five out of 14 of the Health Boards (36% response rate). Two responses (two out of five) were received from Northern Ireland (40% response rate).

### How many Trusts have a service within their own provision for adults, children, or both?

All Trusts who responded in Scotland, Wales and Northern Ireland reported having their own Trust delivered service. For England, 97 out of 104 (93%) responders reported having their own Trust-delivered service, while those with no service signposted to private providers (mostly charities and one council provider). Not all of these Trusts provided NHS low vision services for both children and adults, as can be seen in Table 1.

**Table 1:** shows the number and percentage of responders and levels of provision offered by each of the devolved nations and for the UK as a whole.


	RESPONSE RATE	TRUST-DELIVERED PROVISION	TRUST-DELIVERED PROVISION FOR ADULTS*	TRUST-DELIVERED PROVISION FOR CHILDREN	TRUST-DELIVERED PROVISION FOR CHILDREN & ADULTS*

**England**	104/209 (50%)	97/104 (93%)	91/102 (89%)	85/104 (82%)	79/102 (77%)

**Wales**	6/10 (60%)	6/6 (100%)	6/6 (100%)	6/6 (100%)	6/6 (100%)

**Scotland**	5/14 (36%)	5/5(100%)	5/5(100%)	5/5(100%)	5/5(100%)

**Northern Ireland**	2/5 (40%)	2/2 (100%)	2/2 (100%)	1/2 (50%)	1/2 (50%)

**UK**	117/238 (49%)	110/117 (94%)	104/115 (90%)	97/117 (83%)	91/115 (79%)


*Data excludes the two paediatric-only hospitals in England. In Northern Ireland, neither response was from a paediatric-only hospital, and one reported that the service provided was for adults only.

### Where is the service based?

As expected, the six services in Wales were delivered by NHS-funded provision, (primarily by optometrists in primary care, Welsh Government, 2021). Of the Trust-delivered services in England, Scotland and Northern Ireland (n = 104), the majority were hospital based (n = 76) and a significant number were community based (n = 28) (including two domiciliary services, with 10 Trusts offering both community and hospital services).

### Who leads the service?

Out of the 110 UK-wide respondents who reported having a Trust-delivered low vision service, the low vision services were largely led by either Optometrists (37%) or Orthoptists (32%) or jointly led by both (9%). Other professions leading the services included dispensing opticians (5%), nurses (2%), or an unspecified combination of jointly leading professionals (15%).

### Who works in the Low Vision service?

**Figure 2:**
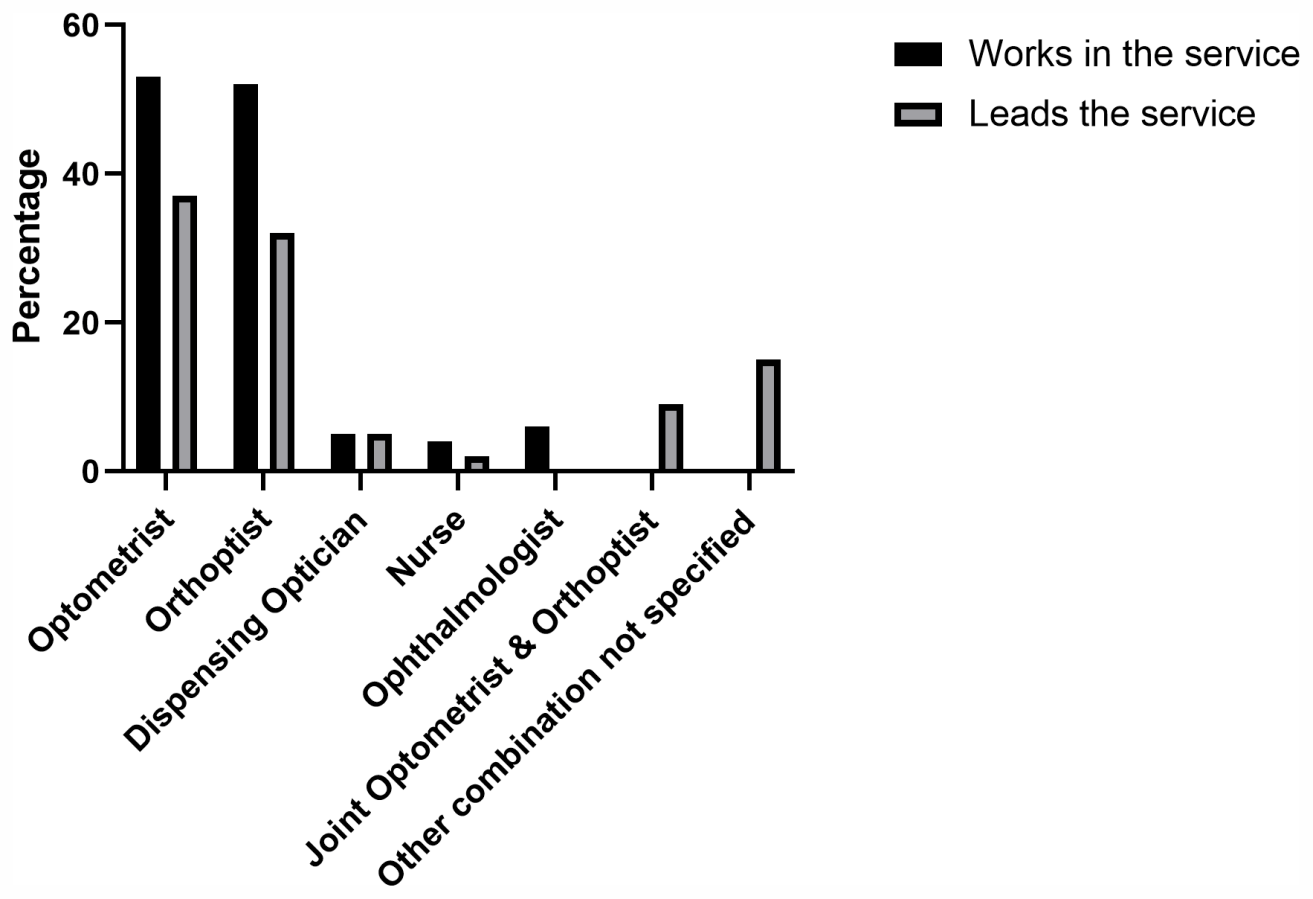
Shows the relative numbers of each profession working within and leading the clinical low vision services, respectively, in percentage.

For Trust-delivered services, the following professional groups were identified as working in the low vision service: Optometrists (n = 58, 53%), Orthoptists (n = 57, 52%); Ophthalmologists (n = 7, 6%), Nurses (n = 4, 4%), dispensing opticians (n = 5, 5%). The majority of Trusts (n = 75) reported having ECLO as part of their Low Vision service. The relative numbers of each profession working within and leading the clinical low vision services can be seen respectively, given in percentages, in Figure 2. Many Trusts employ more than one type of eye-care professional and therefore the percentages of those working in the services do not equate to 100%.

### Which professions offer appointments to children or adults?

**Figure 3:**
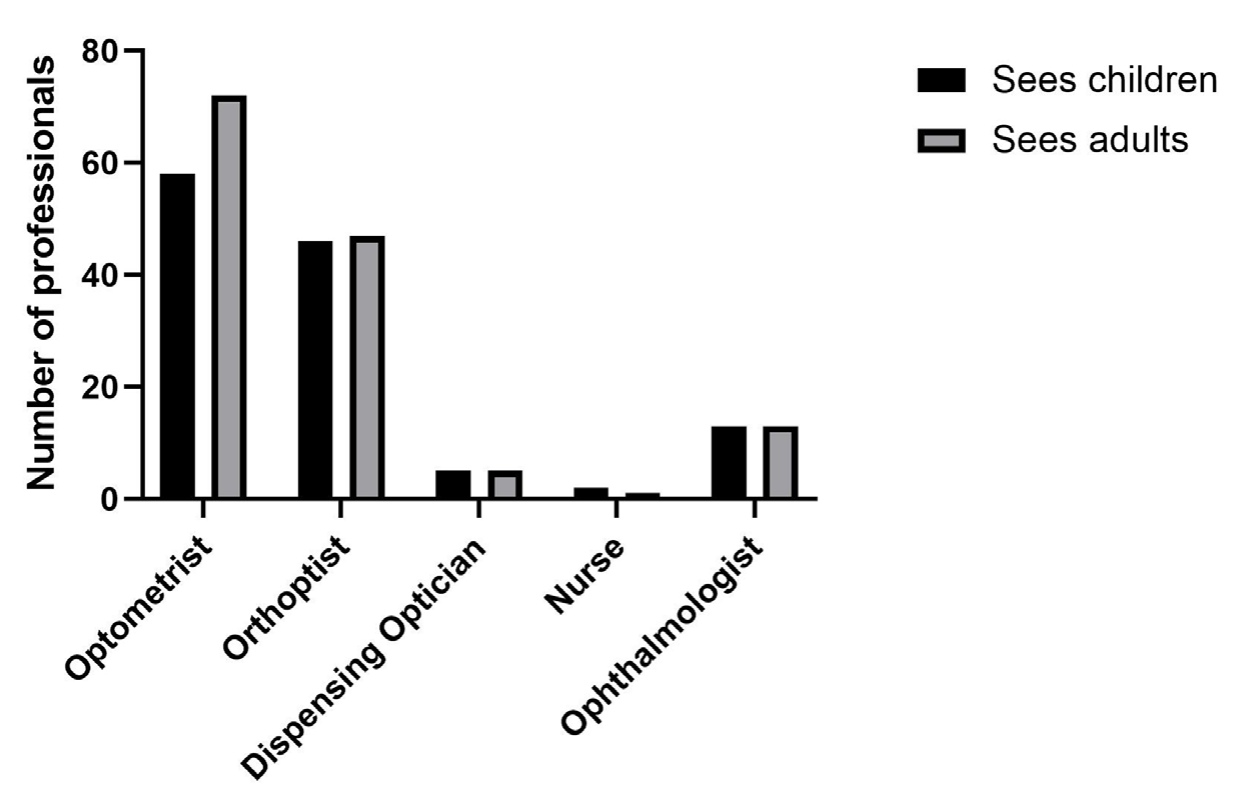
Shows the relative numbers of each profession seeing children and adults, respectively.

Figure 3 shows the relative numbers of each profession offering appointments to children and adults, respectively. Orthoptists, dispensing opticians, and nurses were reported to see children and adults in similar measure. Among all professions, Optometrists were most commonly reported as offering appointments to low vision patients, with slightly more appointments for adults than for children.

## Discussion

The response rates, although relatively good for a survey, were insufficient to fully represent the state of low vision services for children and adults in the UK. Table 1 reports a figure of 79% of the Trusts who responded, providing both adult and paediatric low vision services where applicable (i.e. excluding paediatric hospitals). While it is positive in that the majority of Trusts offer provision to both adults and children, it is important to note that this may potentially overestimate the provision. This is due to the lack of response from 51% of Trusts, leading to a reasonable assumption that Trusts without low vision service provision and specialists involved, would be less likely to respond to the survey invitation.

Whilst the response rate was not ideal, this report identifies is that in some areas, within some Trusts, there is no state funded low vision specialist provision for either children (17% of responding Trusts), adults (10%) or both (21%). For those Trusts without a NHS service, signposting patients to either charity or council provision, the cost to patients is unknown. This evidences the ongoing lack of equality in accessing specialist low vision support and the variation in service and support levels, depending on the location of individuals with VI, as previously reported in by Dickinson et al. in 2011. In some regions, a child with low vision could have access to a specialist low vision practitioner who would liaise with their Qualified Teacher of the Visually Impaired (QTVI), ECLO and signpost to allied services. However, in other regions, a child with similar condition might not have access to a low vision practitioner at all, leaving parents and carers attempting to access provision through charitable or private providers, often at significant distances or costs. Our findings are similar to those reported by Menon et al. (2020), who also suggested that many VI patients receive an inadequate level of information within their appointments.

The majority of Trusts have services which were led by and staffed by Optometrists and Orthoptists. Both professions embrace post-registration education to enhance the specialist skills of their professions in order to facilitate best practice in the area of low vision. Figures 2 and 3 depict the different professions involved in low vision practice, indicating an encouraging trend towards multi-professional teamwork for the benefit of patients. Many Trusts reported having an ECLO working in their low vision service, which is encouraging considering the evidence that ECLOs have a positive impact on the emotional wellbeing of people with VI, and provide practical advice and continuity of care (Menon et al. 2020). We acknowledge the limitation of the questionnaire in not identifying the specific role of the ECLO within the low vision service and whether their role was purely additional support or whether it included other interventions such as low vision aids. The infrastructure and integrated care provided by a Trust-delivered service, allows for input from the multi-disciplinary team (MDT) and collaboration with all eye-care practitioners from primary care through to a tertiary setting (BIOS Low Vision Clinical Advisory Group). The Allied Health Professions (AHP) Strategy for England: AHPs deliver (2022), set out collective priorities and commitments to improve outcomes for patients, carers, communities, and populations. This strategic document has provided a blueprint to assist systems leaders in determining the most effective ways for AHPs to deliver their services in response to evolving care needs. AHPs deliver (2022–2027), encourages AHPs to lead change and deliver evidence-based and informed practice that addresses the variation in service quality and efficiency and overcome service boundaries to reduce fragmentation.

The results found by this study indicate the need for further research that thoroughly analyses low vision service provision, and highlight that it is essential to take steps to diminish the inequalities in provision, access, commissioning and resources in order to ensure patient provision for all. Ideally, a multi-professional research approach is needed, assimilating the responses from Optometry and Ophthalmic nursing colleagues, as well as a more comprehensive Orthoptic response. This report comes at a crucial time in Ophthalmology care, wherein services are stretched more than ever, with Ophthalmology the busiest outpatient department for three consecutive years and most NHS Trusts short of consultant-level care (RCOphth, 2023). There is scope for improvement in patient care, patient outcomes, coordination, and resourcing for low vision provision. This important area of healthcare facilitates improved visual function and increased daily-living independence (Stelmack, et al. 2008), and accessible services for all would offer this benefit more widely.

## Conclusion

Significant variation was found in low vision service provision throughout the UK, with some regions having no NHS-delivered provision for either children, adults, or both. The reported provision, as observed, did not appear to meet patient need equitably and inclusively. Additionally, a significant number of Trusts had unknown provision status, due to the number of non-responders. The Low Vision service-delivery picture in the UK calls for a coordinated response across all professions and healthcare providers, with particular attention to children’s services. We have a timely opportunity to positively drive collaborative, integrated, high quality low vision care. The AHPs strategy-AHPs deliver (2022–2027) encourages AHPs to lead change for evidence-based informed practice to remove service quality and efficiency variation.
